# Gold and Tin Combined as a Filling Material

**Published:** 1885-11

**Authors:** A. W. Harlan

**Affiliations:** Chicago, Illinois


### Independent Practitioner.

VOL. VI. NOVEMBER, 1885. NO. 11.

### Original Communications.

### GOLD AND TIN COMBINED AS A FILLING MATERIAL.

BY A. W. HARLAN, M. D., D. D. S. CHICAGO, ILLINOIS.

READ BEFORE THE CHICAGO DENTAL SOCIETY JULY 1ST, 1884, AND PUBLISHED BY PERMISSION OF THE SOCIETY.

The attentive reader of current dental periodicals cannot have failed to observe the strong tendency on the part of contributors of late to deal with the causes of dental diseases and their appropriate treatment. This tendency has carried with it papers on dental education and legislation. Sparsely intermingling with the latter you may have noticed that a very few writers are engaged in microscopical work, endeavoring to clear up disputed or unsettled facts in dental histology. These efforts have so largely overshadowed the so-called practical subjects, that I have ventured to present to you this evening a paper devoted to a consideration of every-day operations. It will not be disputed, I think, that filling teeth, for a long time to come, will be the chief occupation of all dentists. This being admitted, you will not deem it out of place, I hope, to devote the evening to a discussion of the subject. The writer assumes that gold is, of all materials now in use, the best substance with which to fill the majority of teeth that need to be filled, but it is not practicable to use it in all cases. The reasons why are well known; however, it may be as well to recapitulate them.

First, inability on the part of the patient to pay for such fillings.

Second, lack of physical strength to endure protracted operations.

Third, the impossibility of making fillings uniformly good for the young, the aged and the weak.

Fourth, recognition of the fact that, even though such fillings were always made, they could not be considered permanent on account of the habits, age, or condition of the patient.

Fifth, the absolute folly of filling teeth with gold for the majority of children under the ages of sixteen or eighteen.

Sixth, the certainty of failure to accomplish the desired object, with certain teeth, at all ages beyond the period of childhood.

In addition to the above, every intelligent dentist will encounter cases where his judgment and experience clearly demonstrate at the time, that other materials will be better suited to the case in hand than gold. By this time many of you may be asking this question: What then would you advise us to use, in such cases, in the place of gold? My answer is yet to be made. We have amalgam, tin, Robinson's fibrous material, gutta-percha, oxy-chloride of zinc, oxy-phosphate of zinc, and one other combination of metals, which has not been largely used—gold and tin.

Beginning with amalgam, I would advise it to be used very sparingly, and then only in out-of-the-way places, or in the crowns of large molars. I do not believe that it is good practice to use it in proximal cavities of bicuspids or molars, where the teeth are closely in contact. It is permissible when the cervical wall is well covered with tin or tin and gold. It should not be used in buccal cavities alone, but only where the crown cavity extends through the buccal wall. Use as little as possible between bicuspids. It is generally safe to use amalgam on the proximal surfaces of teeth when the adjacent tooth has been lost. Never use it in a tooth which has been filled with gold, unless the fillings are in contact. In fact, use it very sparingly. It has been used beneath large gold fillings where the teeth have become carious under such fillings. It is just as easy to use tin, and I think much better.

Tin may be used on the buccal surfaces of teeth, and for filling a quarter to one-half of a cavity on the proximal surface of a molar or bicuspid, and for filling cavities in temporary teeth. It may be used in crown cavities in children's permanent molars, and pretty generally for crown cavities in third molars. It is admissible to use it in palatal cavities of incisors, and occasionally in proximal cavities in the same teeth, when not exposed to view.

Gutta-percha may be used, as a rule, in deciduous teeth, but in the permanent teeth it cannot be said that there are many places where it can be called a permanent filling. It will disintegrate on the buccal surfaces, because few persons take proper care of their teeth. It will not stand the wear of mastication for many years. It is not as durable for repairing gold fillings as tin. I use it in incisors occasionally, and on the posterior surfaces of the second molars when the third has been extracted, and rarely elsewhere.

Oxy-chloride of zinc should only be used in pulpless teeth, as a filling material, and then it must be covered with gutta-percha, or a metallic filling. I have seen cases where, in crown cavities in molars, it had been doing good service for from five to eight years. I do not use it in deciduous teeth.

Oxy-phosphate of zinc, as a filling material, is very much like the former, as it cannot be depended upon for permanency. Its chief use is as a capping material, and also for setting artificial crowns. It may be used in the deciduous teeth, and in the first permanent molars, from the sixth to the ninth or tenth years. It can be used in the crowns of molars with or without living pulps, but it should be covered with metal. If I had to choose between oxy-phosphate and oxy-chloride of zinc for proximal cavities in incisors, I would use the former, because it is less irritating. It remains to be seen whether it is more durable in such situations than gutta-percha, or oxy-chloride of zinc. Do not pin your faith to it for a permanent filling.

Robinson's filling, it is claimed, is an excellent material for children's temporary and permanent teeth, as also for lining the cervical margins in proximal cavities, and for filling buccal cavities. My judgment tells me that tin is better, tougher and easier to work under the instrument, and not so liable to turn inky black as is Robinson's metal. I have had two years' experience with it, and have abandoned its use.

The combination of gold and tin is last, but not least, in the consideration of filling materials. Originally used by Dr. F. P. Abbot, of Berlin, the leading American dentist in that city for more than thirty years, it has stood the crucial test. While it may be true that other dentists used gold and tin previous to 1851–5, yet their object was not the same as his; economy in the use of gold was their sole aim. Dr. Abbot, on the contrary, saw that it was not possible to save a certain class of teeth with gold, or amalgam, or tin, alone, and he deliberately commenced to fill teeth by combining gold and tin. I am not conversant with his method of introducing the combined metals, but he must have used it in ropes and cylinders. My method of preparing gold and tin, and the manner of introducing it, I will now explain.

The instruments used are six in number, with large wooden handles. They are well serrated, and are mostly wedge-shaped, or three cornered, having suitable angles, so that all classes of cavities may be reached by them.

Gold and tin is best prepared for use by folding whole or half sheets of Number 4 of each metal with a spatula on a square of spunk, or other suitable material. The gold should not be cohesive. I sometimes fold it so that there are four, eight, twelve, or sixteen thicknesses of the two metals. The gold may be on the external or internal surface, or it may be folded so that one-half of each metal is exposed to view. It does not appear to make any difference in its working quality or durability whether the gold or tin is exposed to the surface. Cylinders are made from the strips in such sizes as are desired. Ropes are made in the same manner that gold ropes are—the gold inside or out, as you choose. They should not be cut into very short pieces, as economy of time is gained by using longer ropes. Gold and tin, when used, should be introduced into cavities not entirely by the wedge principle, as inexperienced workers might cut it up by so doing. For crown and buccal cavities, one piece of rope or folded metal is all that is needed. The middle of the ribbon or rope should be grasped by the pliers, and after reaching the bottom of the cavity the ends should be worked in, one at a time, until the cavity is completely filled. Burnish and finish in the usual manner. For proximal cavities, either cylinders or ropes should be used, as the cavities are more easily and satisfactorily filled by these forms of the combined metals. No special directions are needed by soft gold workers.

I have almost wholly abandoned the use of amalgam since adopting the use of gold and tin. I can commend it for its density, the ease of its insertion, the fine finish it is capable of, its non-conducting and non-shrinkable properties, the compatibility of the combination with tooth structures, and the large variety of cavities which may be filled with it. In addition to the above, the chloride and oxide of tin are antiseptic, which is an advantage. Nitric and hydrochloric acids act readily on tin, but it is well known that neither are ever present in sufficiently large quantities to affect fillings in the teeth. Those who have not tried the combination will, I am sure, be pleased at the rapidity with which such fillings are made. Whenever the dentist finds it desirable to economize for his patient, this combination of metals may be used in buccal, crown, and proximal cavities of molars and bicuspids, generally as far forward as the posterior proximal cavity in the first bicuspid. When not exposed to view, it could be made use of even further forward towards the mesial line. The cavity should be prepared so as to be retentive, and those who have not used it will find it very easy of introduction. I have made quite a number of very large fillings in bicuspids and molars, where the cavities extended from the proximal sur faces into the crowns of such teeth. If the ribbons or ropes are of sufficient length, these fillings are capable of resisting mastication in an astonishing manner. In the majority of cases, where it is manifestly improper to use gold exclusively, gold and tin may be used as quickly as amalgam, and the operator has no fear of there being a leak afterwards. In addition to this, the filling may be finished at the same sitting. Care must be exercised in introducing such fillings, to prevent the point of the instrument from chopping up the material. A little practice out of the mouth will be found advisable by most beginners.

